# Influenza H5N1 virus infection of polarized human alveolar epithelial cells and lung microvascular endothelial cells

**DOI:** 10.1186/1465-9921-10-102

**Published:** 2009-10-30

**Authors:** Michael CW Chan, Renee WY Chan, Wendy CL Yu, Carol CC Ho, WH Chui, CK Lo, Kit M Yuen, Yi Guan, John M Nicholls, JS Malik Peiris

**Affiliations:** 1 Departments of Microbiology, Li Ka Shing Faculty of Medicine, The University of Hong Kong, Queen Mary Hospital, Pokfulam, Hong Kong SAR, PR China; 2 Department of Pathology, Li Ka Shing Faculty of Medicine, The University of Hong Kong, Queen Mary Hospital, Pokfulam, Hong Kong SAR, PR China; 3 Department of Cardiothoracic Surgery, Queen Mary Hospital, Pokfulam, Hong Kong SAR, PR China; 4 Department of Cardiothoracic Surgery, Queen Elizabeth Hospital, Kowloon, Hong Kong SAR, PR China; 5 HKU-Pasteur Research Centre, Hong Kong SAR, PR China

## Abstract

**Background:**

Highly pathogenic avian influenza (HPAI) H5N1 virus is entrenched in poultry in Asia and Africa and continues to infect humans zoonotically causing acute respiratory disease syndrome and death. There is evidence that the virus may sometimes spread beyond respiratory tract to cause disseminated infection. The primary target cell for HPAI H5N1 virus in human lung is the alveolar epithelial cell. Alveolar epithelium and its adjacent lung microvascular endothelium form host barriers to the initiation of infection and dissemination of influenza H5N1 infection in humans. These are polarized cells and the polarity of influenza virus entry and egress as well as the secretion of cytokines and chemokines from the virus infected cells are likely to be central to the pathogenesis of human H5N1 disease.

**Aim:**

To study influenza A (H5N1) virus replication and host innate immune responses in polarized primary human alveolar epithelial cells and lung microvascular endothelial cells and its relevance to the pathogenesis of human H5N1 disease.

**Methods:**

We use an *in vitro *model of polarized primary human alveolar epithelial cells and lung microvascular endothelial cells grown in transwell culture inserts to compare infection with influenza A subtype H1N1 and H5N1 viruses via the apical or basolateral surfaces.

**Results:**

We demonstrate that both influenza H1N1 and H5N1 viruses efficiently infect alveolar epithelial cells from both apical and basolateral surface of the epithelium but release of newly formed virus is mainly from the apical side of the epithelium. In contrast, influenza H5N1 virus, but not H1N1 virus, efficiently infected polarized microvascular endothelial cells from both apical and basolateral aspects. This provides a mechanistic explanation for how H5N1 virus may infect the lung from systemic circulation. Epidemiological evidence has implicated ingestion of virus-contaminated foods as the source of infection in some instances and our data suggests that viremia, secondary to, for example, gastro-intestinal infection, can potentially lead to infection of the lung. HPAI H5N1 virus was a more potent inducer of cytokines (e.g. IP-10, RANTES, IL-6) in comparison to H1N1 virus in alveolar epithelial cells, and these virus-induced chemokines were secreted onto both the apical and basolateral aspects of the polarized alveolar epithelium.

**Conclusion:**

The predilection of viruses for different routes of entry and egress from the infected cell is important in understanding the pathogenesis of influenza H5N1 infection and may help unravel the pathogenesis of human H5N1 disease.

## Introduction

Highly pathogenic influenza (HPAI) H5N1 virus first emerged as a cause of severe human disease in 1997 in Hong Kong [1,2]. Since then, it has become entrenched in poultry across Asia and Africa with zoonotic transmission to humans, sometimes with fatal outcome. In contrast to human seasonal influenza, H5N1 disease has a higher reported case-fatality rate ranging from 33% in Hong Kong in 1997 to 61% more recently [1,3]. The reason for this unusual severity of human disease remains unclear. Within the lung, the alveolar epithelium is the primary target cell for influenza H5N1 virus [4-6]. Although a novel influenza H1N1 virus of swine origin has recently emerged to cause a pandemic [7,8], the pathogenesis of H5N1 virus remains an important public health issue because this virus remains a pandemic and public health threat, either directly or through reassortment with the novel pandemic H1N1 virus.

Epithelial cells line the major cavities of the body, functioning in selective secretion and adsorption, and providing a barrier to the external environment. In human lung, the alveolar epithelium consists of a continuous layer of tissue made up of two principal cell types: flattened type I alveolar epithelial cells and cubodial type II alveolar epithelial cells. Type I alveolar epithelial cells cover over 80% of the alveolar surface in which they function as a broad thin layer for gaseous exchange. These cells are highly polarized since the plasma membranes of these cells are divided into two discrete domains, namely, the apical domain (facing the luminal air surface) and the basolateral domain (facing the systemic circulation) [9]. And this large thin surface makes them extremely susceptible to injury from inhaled pathogens. While there is some data on H5N1 virus infection and cytokine responses in alveolar epithelial cells [10], there is no information of the effect of cell polarity on H5N1 virus replication or on virus-induced host responses.

Though influenza virus infection is localized primarily to the respiratory system, HPAI in some avian species is associated with systemic dissemination of the virus to multiple organs. There is increasing evidence that H5N1 influenza viruses are found in the peripheral blood, the gastro-intestinal tract and occasionally even the central nervous system of humans, and such dissemination may contribute to unusual disease manifestations including those of multiple organ dysfunction [11-14]. The close anatomical relationship between alveolar epithelium and the lung microvascular endothelium, together with the distribution of putative influenza A virus receptors on the endothelial cell surface [15], make it important that parallel investigations on the lung epithelium and lung endothelium are carried out.

In the present study, we investigated the infection of polarized, primary, human type I-like alveolar epithelial cells and lung microvascular endothelial cells by influenza A virus. The low pathogenic human seasonal influenza virus, A/HK/54/98 (H1N1) and the HPAI A/HK/483/97 (H5N1) virus were studied. We found that both influenza H1N1 and H5N1 viruses efficiently infect alveolar epithelial cells from both apical and basolateral surface of the epithelium. Irrespective of the route of infection, both viruses were preferentially released at the apical surface of the alveolar epithelium. Whereas in lung microvascular endothelial cells, influenza H1N1 virus failed to replicate convincingly in contrast, influenza H5N1 virus showed evidence of replication following infection by either apical or basolateral route and new virus was released from both sides of the cell. As previously reported [10], influenza H5N1 virus was a more potent inducer of cytokines and chemokines (e.g. IP-10, RANTES, IL-6) in comparison to H1N1 virus in alveolar epithelial cells. Influenza H5N1 virus induced chemokines were secreted on both the apical and basolateral aspects of the polarized alveolar epithelium while the human influenza H1N1 virus led predominantly to apical secretion. These findings enhance the understanding of how virus infection may spread within and beyond the lung in influenza virus infection and how innate host response may contribute to modulating or aggravating tissue pathology.

## Materials and methods

### Isolation of primary human alveolar epithelial cells

Primary alveolar epithelial cells were isolated from human non-tumor lung tissue obtained from patients undergoing lung resection in the Department of Cardiothoracic Surgery, Queen Mary Hospital and Queen Elizabeth Hospital, Hong Kong SAR, under a study approved by The Hong Kong University and Hospital Authority (Hong Kong West and Kowloon Central/East, respectively) Institutional Review Board, using a modification of methods previously described [16]. Briefly, lung tissue was minced into pieces of > 0.5 mm thickness using a tissue chopper. The tissue was digested using a combination of trypsin and elastase for 15 min at 37°C in a shaking water-bath. The cell population was purified by a combination of differentiated cell attachment, Percoll density gradient centrifugation and magnetic cell sorting. The cells were maintained in a humidified atmosphere (5% CO_2_, 37°C) under liquid-covered conditions, and growth medium was changed daily starting from 60 h after plating the cells.

### Type I-like alveolar epithelial cell differentiation and polarization

The purified cell pellet (passage 1 or 2) was resuspended in medium to a final concentration that allowed seeding at 5 × 10^5 ^cells/cm^2 ^onto collagen I coated Transwell supports (Corning) and cultured for 14 to 20 days with the small airway culture medium SAGM (Lonza) in the apical and basolateral chambers of the Transwell. The cells spread to form a confluent monolayer and the culture medium was changed every 48 h. A concomitant increase in transepithelial electrical resistance (TER) was measured using an epithelial tissue voltohmmeter (EVOM). TER was calculated as the measured electrical resistance (Ohms) multiplied by the surface area of the filter. This method has already been established in our laboratory using a modification of the methods previously described [16,17]. When the transepithelial electrial resistance (TEER) reached 1000 ohm cm^2^, which demonstrate the paracellular restrictiveness of the alveolar cell preparation, the competence of the formation of tight junctional complexes within the polarized alveolar epithelial cells model can be assessed [16] and the cells were used for virus infection experiments.

### Culture and polarization of lung microvascular endothelial cell

Primary human lung microvascular endothelial cells (HLMVE) were purchased from Lonza Walkersville, Inc. (US) and maintained in the medium and growth supplements supplied by the manufacturer (EGM-2), which contained 5% fetal bovine serum (FBS), hydrocortisone, human endothelial growth factor, vascular endothelial cell growth factor, human fibroblast growth factor basic, long(R3)-insulin-like growth factor-1, ascorbic acid and antibiotics. Medium was changed every 48 h until confluence. The HLMVE were seeded in the apical compartment of a 0.4 μm pore size transwell support (Corning) with a cell density of 5 × 10^5 ^cells/cm^2^. The cells were cultured for 10 days with medium changed in both the apical and basolateral compartments every 48 h. When the transepithelial electrial resistance (TEER) reached 25 ohm cm^2^, the cells were used for virus infection experiment [18].

### Viruses

We used HPAI H5N1 virus (A/Hong Kong/483/97), a virus isolated from a patient with fatal influenza H5N1 disease in Hong Kong in 1997, and A/Hong Kong/54/98 (H1N1) as a representative seasonal influenza virus, for our comparative studies. Viruses were initially isolated and subsequently maintained in Madin-Darby canine kidney (MDCK) cells. They were cloned by limiting dilution and seed virus stocks were prepared in MDCK cells. Virus infectivity was assessed by titration of tissue culture infection dose 50% (TCID_50_) in MDCK cells. The influenza H5N1 virus used in this study was handled in a Bio-safety level 3 (BSL-3) facilities in the Department of Microbiology, The University of Hong Kong.

### Virus infection of cells

**Figure 1:**
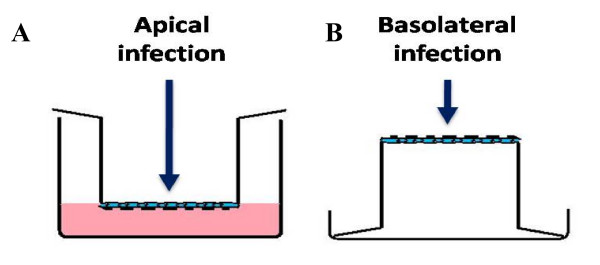
**Representation of the transwell insert-setup during the influenza virus infection experiment**. Type I-like alveolar epithelial cells or HLMVEs (indicated in blue) were seeded on top of the porous membrane. During apical infection (A) virus was added into the apical compartment while the transwell was placed upside down during basolateral infection (B).

Virus inoculation procedures were designed to determine the role of cell polarity in direction of infection, virus release and cytokine secretion. Polarized type I-like alveolar epithelial cells and HLMVE were seeded on the apical surface of the transwell membrane and infected from the apical or basolateral surface respectively. During apical infection, 200 μl of virus was added into the apical compartment of the transwell (Figure 1A) while during basolateral infection, 80 μl of virus was added onto the transwell membrane with the transwell oriented upside down (Figure 1B). The orientation of the transwell in the apical infection situation was resumed at 1 h after virus inoculation and the washing steps. In this series of experiments, we used a MOI of 0.01 to evaluate the difference between the two routes of infection in terms of release of newly formed virus and at MOI of 2 to determine the percentage of cell infection and cytokine release.

### Virus replication analysis

Evidence of viral infection was established by a) assaying viral matrix RNA at 1, 3, 6 and 24 h post infection by quantitative RT-PCR, b) viral antigen expression by immunofluorescence staining with mouse anti-influenza nucleoprotein and matrix antibody conjugated with FITC (DAKO Imagen, Dako Diagnostics Ltd, Ely, UK) and c) assaying infectious virus in cell culture supernatant by TCID_50 _assay to demonstrate complete virus replication.

### Quantification of cytokine and chemokine mRNA by real-time quantitative RT-PCR

DNase-treated mRNA from infected cells model was extracted at 1, 3, 6 and 24 h post infection using RNeasy Mini kit (Qiagen, Hilden, Germany). The cDNA was synthesized from mRNA with Oligo-dT primers and Superscript III reverse transcriptase (Invitrogen) and quantified by real-time quantitative PCR analysis with a LightCycler (Roche, Mannheim, Germany). The gene expression profile for cytokines (interferon beta (IFN-β), IL-6) and chemokines (IP-10, RANTES) and viral matrix gene were quantified and normalized using the housekeeping gene product β-actin mRNA.

### Quantification of cytokine and chemokine proteins by ELISA

The concentrations of IP-10, RANTES, IL-6 and IFN-β proteins in the influenza virus infected type I-like alveolar epithelial cells were measured by a specific ELISA assay (R&D Systems, Minneapolis, MN, USA). Samples of culture supernatant were irradiated with ultraviolet light (CL-100 Ultra Violet Cross linker) for 15 min to inactivate any infectious virus before the ELISA assays were done. Previous experiments had confirmed that the dose of ultraviolet light used did not affect cytokine concentration as measured by ELISA (data not shown).

### Lectin histochemistry

Type I-like alveolar epithelial cells cultured in transwell insert and HLMVE cell pellet were fixed with 10% formalin and sectioned at 5 μm followed by lectin histochemistry as published previously [19]. The cells were microwaved in 10 mM citrate buffer pH 6.0 at 95 °C for 15 min then blocked with 3% H_2_O_2 _in TBS for 12 min and with avidin/biotin blocking kit (Vector). They were then incubated with 1:100 HRP conjugated *Sambucus nigra *agglutinin (SNA) (EY Laboratories), 1:100 biotinylated MAL-I and MAL-II (Vector) and Digoxigenin conjugated MAA (Roche) for 1 h at room temperature (RT), blocked with 1% bovine serum albumin for 10 min at RT, and then incubated with strep-ABC complex (Dako Cytomation, K-0377) diluted 1:100 for 30 min at RT. Development was performed using the AEC substrate kit (Vector) at RT for 10 min, the nuclei were counterstained with Mayer's hematoxylin and then the sections were dried and mounted with DAKO aqueous mount (Dako Cytomation). Duck intestine sections were used as controls with and without pre-treatment with sialic acid (Sia) α2-3 specific neuraminidase from Glyko to ensure that Sias were specifically targeted.

### Statistical analysis

Two-tailed student *t*-test was used to compare the different of viral titers in the influenza virus infected cell supernatants between the early and late time point. The quantitative cytokine and chemokine mRNA and protein expression profile of mock, influenza H1N1 and H5N1 virus infected cells were compared using one-way ANOVA, followed by Bonferroni multiple-comparison test. Differences were considered significant at *p *< 0.05.

## Results

### Sialic acid receptor distribution on the polarized type I-like alveolar epithelial cell and HLMVE

**Figure 2:**
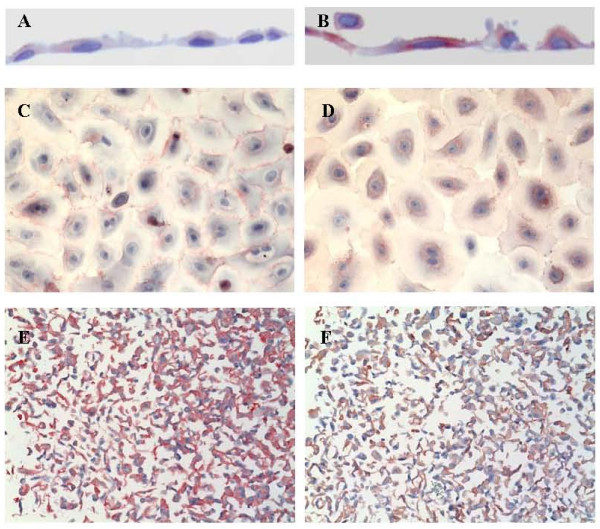
**Lectin binding assay to determine the Sias distribution on polarized type I-like alveolar epithelial cells and HLMVEs**. (A, C, E) SNA binds to Siaa2-6Gal and (B, D, F) MAA binds to Siaa2-3Gal presented on the (A-D) type I-like alveolar epithelial cells and (E-F) HLMVE cell pellet in reddish brown. *En *face sections were generated from selected planes in the vertical sections.

Lectin histochemistry on the primary culture of human type I-like alveolar epithelial cells using SNA and MAA showed that MALII (which recognizes the accepted avian influenza receptor Siaa2-3Galβ1-3GalNAc) bound strongly to the type I-like alveolar epithelial cells (Figure 2A-2D). Staining with SNA which recognizes the human influenza receptor Siaα2-6 in type I-like alveolar epithelial cells was not prominent on the type I-like alveolar epithelial cells, results that are similar to the report by Shinya *et al *[5]. The lectin histochemistry on the HLMVE cells shows binding of both SNA (Figure 2E) and MALII (Figure 2F) which agrees with previous reports [15].

### Polarity of influenza A virus infection and replication in alveolar epithelial cells

**Figure 3:**
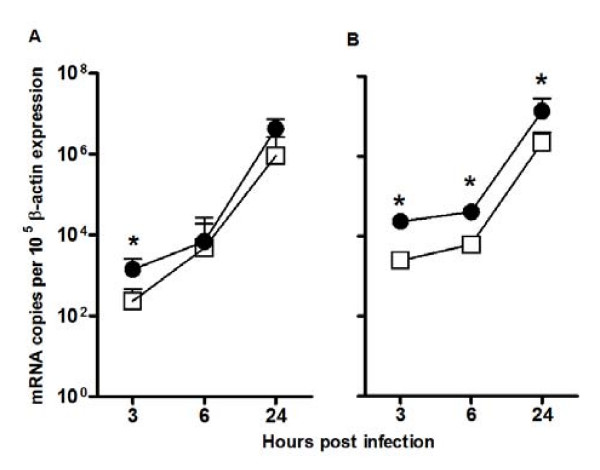
**Viral matrix (M) gene expression in copy number normalized with β-actin gene expression (10^5 ^copies) during infection of human primary type I-like alveolar epithelial cell**. M gene expression in type I-like alveolar epithelial cells infected (MOI = 2) with influenza (A) A/HK/54/98 (H1N1) and (B) A/HK/483/97 (H5N1) virus. Black circles indicate gene expression after apical infection and open square indicates gene expression after basolateral infection. Asterisk indicates a greater M gene expression in apical infected cells than basolateral infected cells with statistical significance of *p *< 0.05.

Both influenza A viruses, A/HK/54/98 (H1N1) and A/HK/483/97 (H5N1) were able to infect the type I-like alveolar epithelial cells from both the apical and basolateral compartments. The influenza matrix gene expression increased from 3 h to 24 h post infection (*p *< 0.05). Apical infection resulted in greater matrix gene copy number at 3 h post infection compared with basolateral infection of type I-like alveolar epithelial cells with A/HK/54/98 (H1N1) but these differences were less marked at 6 h and 24 h post infection. The matrix gene copy number in type I-like alveolar epithelial cells infected with influenza A/HK/483/97 (H5N1) virus apically was higher than that of cells infected via the basolateral aspect at all three time points, 3 h, 6 h and 24 h post infection (Figure 3).

**Figure 4:**
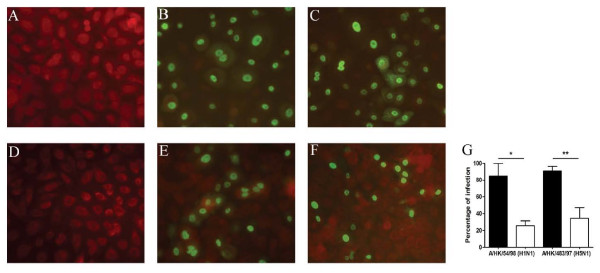
**A representative immunofluorescence staining of type I-like alveolar epithelial cells after (A, D) mock, (B, E) influenza H1N1 and (C, F) H5N1 virus infection and (G) chart with percentage of infection**. Virus matrix protein and nucleoprotein were stained green by FITC-conjugated mouse antibody. The immunofluoresecent staining of the type I-like alveolar epithelial cells after apical (A-C) and basolateral (D-F) infection at 24 h post infection respectively. (G) Bar chart shows the mean percentage of infection and error bar represent the standard derivation, dark bar represents apical infection and open bar represents basolateral infection. Single and double asterisk indicates statistically significant difference with *p *< 0.05 and *p *< 0.01 respectively.

Viral protein expression was detected by immunofluoresence in both influenza A/HK/54/98 (H1N1) and A/HK/483/97 (H5N1) virus infected alveolar epithelial cells, infected from the apical or basolateral aspect. The number of cells infected was significantly higher (*p *= 0.02 and *p *= 0.01) in influenza A/HK/54/98 (H1N1) and A/HK/483/97 (H5N1) virus, respectively when the virus was inoculated from the apical than from the basolateral side (Figure 4).

**Figure 5:**
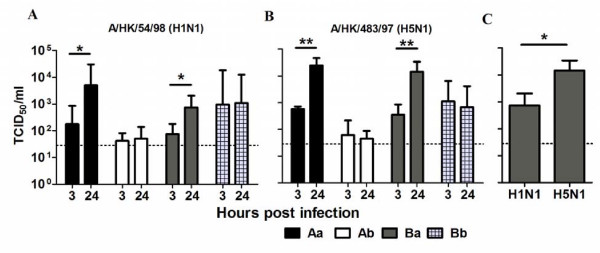
**Virus titer detected in the supernatant of influenza virus infected type I-like alveolar epithelial cells**. Virus titer of the (A) A/HK/54/98 (H1N1) and (B) A/HK/483/97 (H5N1) was determined after apical infection and basolateral infection of the type I-like alveolar epithelial cells at 3 h and 24 h post infection. Aa = apical release after apical infection, Ab = basolateral release after apical infection, Ba = apical release after basolateral infection, Bb = basolateral release after basolateral infection. (C) At 24 h post infection following basolateral infection of type I-like alveolar epithelial cells, the titers of influenza H5N1 virus at the apical aspect of the cells is significantly more seen with H1N1 infected cells. Single and double asterisk indicates statistically significant difference with *p *< 0.05 and *p *< 0.01, respectively. Dotted line represents the lowest detection limit of the TCID_50 _assay.

TCID_50 _assay was performed on the supernatant collected from the infected type I-like alveolar epithelial cells infected with highly pathogenic influenza H5N1 (A/HK/483/97) or low pathogenic human influenza H1N1 (A/HK/54/98) viruses to determine the infectious virus yield. Regardless of the infection route and virus strain, increasing TCID_50 _titers were observed in supernatants collected from the apical compartment but not from the basolateral compartment (Figure 5, legend Ab and Bb) (Figure 5). Release of newly formed virus from type I-like alveolar epithelial cells infected via the apical or basolateral surface by either influenza H1N1 or H5N1 viruses was restricted to the apical side of the cells (Figure 5A and 5B). Following basolateral infection, the titers of virus shed on the apical aspect of the cell was higher (*p *= 0.017) following influenza H5N1 virus infection rather than influenza H1N1 virus infection (Figure 5C).

### Polarity of influenza virus infection and replication in lung microvascular endothelial cell

**Figure 6:**
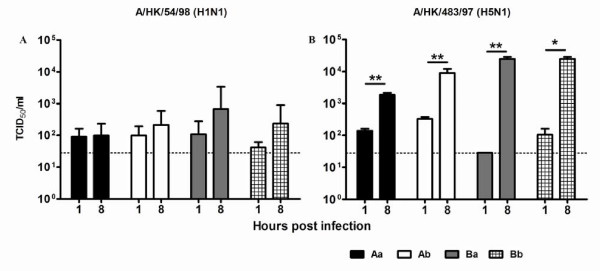
**Virus titer detected in the supernatant of influenza virus infected HLMVE cells**. Virus titer of the (A) A/HK/54/98 (H1N1) and (B) A/HK/483/97 (H5N1) was determined after apical infection and basolateral infection of the HLMVE cells at 1 h and 8 h post infection. Aa = apical release after apical infection, Ab = basolateral release after apical infection, Ba = apical release after basolateral infection, Bb = basolateral release after basolateral infection. Single and double asterisk indicates statistically significant difference with *p *< 0.05 and *p *< 0.01 respectively. Dotted line represents the lowest detection limit of the TCID_50 _assay.

In order to better understand the implications of the effects of cell polarity on virus infection in relation to virus dissemination via the systemic circulation, we investigated the replication of influenza H5N1 and H1N1 viruses in polarized HLMVE cells. There was no convincing evidence of influenza H1N1 virus replication when HLMVE cells were infected via either the apical or basolateral aspect (Figure 6A). Interestingly, HLMVE cells infected with influenza H5N1 virus via either apical or basolateral aspect resulted in virus release from both apical and basolateral aspects of the cell (*p *< 0.05) (Figure 6B).

### Expression of cytokine and chemokine in type I-like alveolar epithelial cell infected with influenza virus through apical and basolateral routes

**Figure 7:**
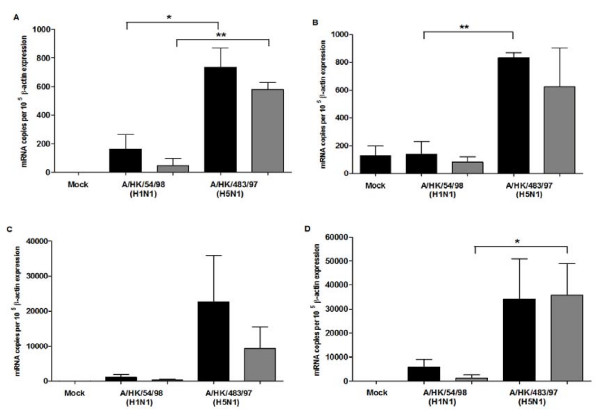
**Cytokine and chemokine gene expression in type I-like alveolar epithelial cells after influenza virus infection**. The cytokine (A) IFN-β, (B) IL-6 and chemokine (C) RANTES, (D) IP-10 gene expression from type I-like alveolar epithelial cell after apical (black) and basolateral (grey) influenza A virus infection at 24 h post infection. The graph shows the mean and the standard error from three representative experiments. Single and double asterisk indicates statistically significant difference with *p *< 0.05 and *p *< 0.005 respectively.

We next investigated the effects of cell polarity on cytokine and chemokine induction by influenza H1N1 and H5N1 virus infected primary human type I-like alveolar epithelial cells. Specifically, we wanted to determine whether the apical and basolateral infection route led to qualitative or quantitative differences in the profile of cytokines induced. The efficiency of infection of the cells by the apical route was 70-100% and basolateral route was 30-50%. As previously reported by us, there was a trend that influenza H5N1 virus infection led to increased levels of cytokine mRNA at 24 h post infection when compared with influenza H1N1 virus, irrespective of whether such infection occurred by the apical (black bars) or basolateral (grey bars) aspect (Figure 7). The differences between influenza H5N1 and H1N1 viruses achieved statistical significance with IFN β following apical (*p *< 0.05) and basolateral (*p *< 0.01) infection (Figure 7A), IL-6 following apical infection (*p *< 0.01) (Figure 7B), and IP-10 following basolateral infection (*p *< 0.05) (Figure 7D). While there was a trend suggesting that the chemokine gene RANTES was hyper-induced by influenza H5N1 virus when compared to that in influenza H1N1 virus, statistical significance was not achieved (*p *= 0.08 in apical infection and *p *= 0.14 in basolateral infection (Figure 7C). Similar cytokine and chemokine expression profiles were observed at 3 h and 6 h post infection in influenza virus infected type I-like alveolar epithelial cells (data not shown). Inactivation of the virus by ultraviolet irradiation prior to infection of the type I-like alveolar epithelial cells abolished cytokine induction (data not shown) suggesting that virus replication was required for cytokine induction. Furthermore, even an increase in the MOI of influenza H1N1 virus up to 5 did not result in the cytokine mRNA expression level to levels similar to those induced by influenza H5N1 virus (data not shown). Broadly, the data from the apical infection are consistent with our previous finding the differential induction of proinflammatory cytokine by influenza H5N1 virus in alveolar epithelial cells [10]. We now have thus confirmed that these differences also apply in polarized alveolar epithelium following infection via the basolateral aspect.

### Polarity of cytokine secretion in influenza H5N1 virus infected alveolar epithelium

**Figure 8:**
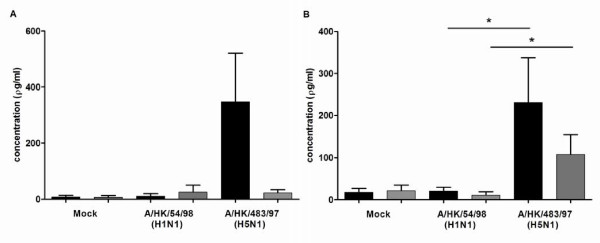
**Chemokine secretion from type I-like alveolar epithelial cells after influenza virus infection**. The apical (dark bar) and basolateral release (grey bar) of (A) RANTES and (B) IP-10 protein from type I-like alveolar epithelial cell after apical infection of A/HK/54/98 (H1N1) and A/HK/483/97 (H5N1). Single asterisk indicates statistically significant difference with *p *< 0.05.

We next investigated whether there was polarity in the secretion of cytokine proteins from type I-like alveolar epithelial cells infected by influenza H1N1 and H5N1 viruses. The concentrations of the IP-10, RANTES and IFN β were measured by ELISA in apical and basolateral culture supernatants of type I-like alveolar epithelial cells infected by the apical route. In parallel with the gene expression profile, influenza H5N1 virus elicited more chemokine release in type I-like alveolar epithelial cells than influenza H1N1 virus, at 24 h post infection. Influenza H5N1 virus induced IP-10 protein secretion was found on the apical side of the polarized type I-like alveolar epithelial cells. This level was significantly higher than the mock infected cells (*p *< 0.01) and influenza H1N1 virus infected cells (*p *< 0.05). In addition, a significantly more IP-10 was secreted from the basolateral side of the influenza H5N1 virus infected alveolar epithelial cells when compared to mock and influenza H1N1 virus infected cells (*p *< 0.05). In contrast, RANTES appeared only to be secreted on the apical aspect of influenza H5N1 virus infected type I-like alveolar epithelial cells although these results did not achieve statistical significance (Figure 8A). We failed to detect any IFN β proteins in the supernatant of type I-like alveolar epithelial cells after influenza virus infection (data no shown) but it should be noted that the limit of detection of the IFN β ELISA was high (250 pg/ml) and this lack of sensitivity of the assay is likely to be responsible for this lack of detection.

## Discussion

In this study, we compared human influenza H1N1 virus with a highly pathogenic influenza H5N1 virus to investigate whether there are differences in the polarity of virus infection and of host cytokine responses in human polarized type I-like alveolar epithelium. The basolateral aspect of the alveolar epithelium lies in close proximity to the basolateral aspect of the lung microvascular endothelial cells raising the question of whether virus regressing the basolateral aspect of the type I-like alveolar epithelial cells can infect the lung microvascular endothelial cells by the basolateral aspect. Alternatively, since influenza H5N1 virus is believed to disseminate systemically and has been detected in the peripheral circulation, it is relevant to understand whether endothelial cells can be infected via the apical aspect, thereby allowing virus in the blood circulation to infect these cells and traffic outward to infect the lung alveolar epithelium from the basolateral aspect. As the lung endothelium covers about 20% of the total surface area of the alveoli sac, the rest being covered by the alveolar epithelium [20], the tropism of influenza A virus in both alveolar epithelium and endothelium is important in the pathogenesis of influenza H5N1 virus infection in human.

Previously, the polarity of influenza virus infection and release have only been studied with low pathogenic influenza A virus (subtype H3N2) and low pathogenic avian influenza A virus (subtype H5N3 and H4N6) [21] in human airway trachea-bronchial epithelial cells. It was demonstrated that newly forming influenza virus was released from the apical surface of respiratory epithelium [21,22]. However, the mouse-adapted influenza H1N1 virus (WSN strain) and Sendi virus have been shown to bud from the apical and basolateral domains [23,24]. Vesticular stomatitis virus and retroviruses [25] are released from the basolateral domain of polarized cells, the symmetrical or asymmetrical binding, internalization and budding of virus from polarized epithelial cells in culture therefore would have potential implications for viral pathogenesis. For example, with coronavirus, the apical release of transmissible gastroenteritis virus resulted in a local infection *in vivo *while the basolateral release of mouse hepatitis virus (MHV) in epithelial cells resulted in systemic infection [26]. In mouse studies, MHV initially replicates in the nasal epithelium before being disseminated throughout the body. The basolateral release of MHV from epithelial cells into the animal's circulation was postulated as the first step in the establishment of a systemic infection.

We showed that both influenza H1N1 and H5N1 viruses preferentially infect type I-like alveolar epithelial cell from its apical surface with higher levels of viral M gene expression as well as higher percentages of cells being infected, when compared to basolateral infection (Figure 3 and 4). This is expected since respiratory viruses need to be adapted to efficiently infect cells via the apical surface, which is the surface that is exposed to the respiratory lumen, and therefore accessible to infection. With influenza H1N1 virus, release of newly formed virus was restricted to the apical aspect, irrespective of whether the alveolar epithelial cells were infected by the apical or basolateral route (Figure 5). Again this is expected with a virus that appears not to disseminate beyond the lung. Similar observations have been reported in parainfluenza virus infected epithelia with the virus preferentially entering and exiting via the apical surface [27,28]. Given its propensity to disseminate beyond the lung, we initially hypothesized that influenza H5N1 virus may be released from both apical and basolateral aspects. But this proved not to be the case and H5N1 was similar to H1N1 in this respect, i.e. virus was released predominantly via the apical aspect, irrespective of the route of infection of the cell (Figure 5B). While the efficiency of infection via the basolateral aspect was lower than that from the apical aspect for both viruses, cells infected with H5N1 virus via the basolateral aspect resulted in a greater than 10 fold higher virus yields on the apical surface than cells comparably infected with H1N1.

We then investigated virus replication in polarized lung microvascular endothelium which is anatomically in close proximity to the alveolar epithelium. There was no convincing evidence of replication of H1N1 virus in the polarized HLMVE cells (Figure 6A). In contrast, H5N1 virus could initiate productive replication of these cells from either aspect and virus release also occurred from apical or basolateral aspect of the cell (Figure 6B). Although neither influenza H5N1 nor H1N1 viruses are efficiently released via the basolateral aspect of the alveolar epithelium, virus replication is likely to lead to weakening of the tight-junctions and to cell death, thus providing these viruses access to the underlying tissues and the basolateral aspect of microvascular endothelial cells. As the lung microvascular endothelium also comprises 20% of the total surface area of the alveoli [20], influenza virus entry via the basolateral aspect of HLMVE cells, replication within them and release from the apical aspect of these cells could lead to viremia and dissemination of infection. The fact that HPAI H5N1 virus H0 precursor form can be cleaved by proteases not restricted to the lung [29] facilitates disseminated virus infection.

The fact that influenza H5N1 (but not H1N1) virus can infect the HLMVE cells from the basolateral aspect would facilitate dissemination of this virus via the blood stream. Furthermore, the observation that HLMVE cells can be infected via the apical aspect and release virus from the basolateral aspect (as well as the apical side) suggests that systemically circulating virus can initiate infection in the lung parenchyma via the endothelial route. This is particularly relevant since recent studies in mice have shown that HPAI H5N1 virus experimentally injected into muscle can led to fatal virus infection with virus establishing infection in the lungs and brain [30]. Furthermore, there has been speculation and anecdotal evidence that H5N1 virus can initiate infection via ingestion and the gastrointestinal tract [31]. The possibility that virus in the systemic circulation can establish a foothold in the lung is therefore an important observation.

Infected type I alveolar epithelial cells undergo either cytolytic or apoptotic death. The shedding of infected type I alveolar epithelial cells may further the inflammation and the underlining interstitial cells may then be exposed to the alveolar lumen fluid which contains high concentrations of virus. Reconstitution of the alveolar epithelial surface depends on the regeneration type I alveolar epithelial cell from its progenitor - the type II alveolar epithelial cells [32]. However, an intact basement membrane is essential for epithelial cell proliferation to occur. Alveolar basement membrane with denuded alveolar epithelial cell will accelerate the epithelial proliferation until the epithelial layer becomes confluent [33]. Nevertheless, type II alveolar epithelial cells could dominate the epithelial surface and prevent the reappearance of type I alveolar epithelial cell when injury signal of type I alveolar epithelial cells persists in the microenvironment [34].

Previous reports on human lung epithelial cell line A549 infected with human influenza H3N2 virus showed a low production of interferons and TNF-α [35]. We have previously shown, compared with influenza H1N1 virus, influenza H5N1 virus differentially upregulated cytokine and chemokine gene expression in alveolar epithelial cells [10] and macrophages [36]*in vitro *experiments and that the profile of differentially upregulated cytokines corresponds with the elevated IP-10 and MIG levels of H5N1 patients serum [37]. Interestingly, IP-10 and MIG have been reported to play roles in the pathogenesis of tissue necrosis and vascular damage associated with certain EBV-positive lymphoproliferative processes [38]. These results again may dictate the different pathogenesis of the downstream cytokine and chemokine response events and contribute to the unusual adverse pathology in H5N1 patient.

This study is the first demonstration of polarity secretion of cytokines in influenza H5N1 infected alveolar epithelium. The secretion of chemokines, notably IP-10, from both the apical and basolateral aspect, was found in influenza H5N1 virus infected type I-like alveolar epithelial cell but not in influenza H1N1 virus infected cell. This could potentially be relevant to the pathogenesis of influenza H5N1 virus infected patients. The basolateral release of chemotactic IP-10 from the influenza virus infected type I-like alveolar epithelial cells recruit lymphocytes from the capillary circulation into alveoli. The binding of chemokines to the receptor of the recruiting leukocytes is specific and leads to a rapid change in the cell shape and behavior of the subpopulation of the leukocyte. This makes them capable of migrating from the blood through the vascular endothelium into the site of inflammation [39,40]. Previous studies on IP-10 and transendothelial migration indicated that IP-10 retained on endothelial cells could induce transendothelial chemotaxis of activated T cells [41]. Another investigation on the biological activity of human recombinant IP-10 investigation further verified its chemotactic properties towards human peripheral blood monocytes and stimulated human peripheral blood T lymphocytes, but not neutrophils [42,43]. One of the studies used endothelial cell adhesion assay to demonstrate the effect of IP-10 in potentiating T cell adhesion to endothelium [43]. The potent secretion of IP-10 from both apical and basolateral side of the infected alveolar type I-like alveolar epithelial cell that we observed would suggest a possible directional recruitment and hence, migration of T cells and monocytes from the lung blood capillaries through microvascular transendothelial migration. Thus macrophages, differentiated from the recruited monocytes, may dominate the alveolar space [13,17,37] and T lymphocytes may occupy the interstitial space [13,17,37,44,45] as previously documented in autopsy reports of patients dying with influenza H5N1 virus infection. As cytokine secreting macrophages accumulate within the alveoli, further augmentation of the cytokine and chemokine cascades may result. Since influenza H5N1 virus is reportedly resistant to the anti-viral effects of interferons and TNF-α [46] and can lead to delayed apoptosis of infected macrophages [47], the clearance of the virus and lung inflammation would take a longer period of time than with seasonal influenza infection. Such prolonged inflammation would eventually result in pathological features with diffuse alveolar damage, hemorrhage [48] and finally interstitial fibrosis [13,45,49,50], which are some key observations in the H5N1 patients.

## Conclusion

In this study, we demonstrate that both influenza H1N1 and H5N1 viruses efficiently infect alveolar epithelial cells from both apical and basolateral surface of the epithelium but release of newly formed virus is mainly from the apical side of the epithelium. In contrast, influenza H5N1 virus, but not influenza H1N1 virus, efficiently infected polarized lung microvascular endothelial cells from either apical or basolateral aspect and also be released from either aspect of these polarized cells. This is likely to be of relevance to the pathogenesis and provides a possible explanation for the entry to the respiratory tract via the blood stream, as proposed by some who suggest that the gastro-intestinal tract can be a portal of entry for this virus. In addition, the release of inflammatory mediators such as IP-10 may be important contributors to the pathogenesis of the disease. More detailed studies on the mechanisms of alveolar epithelial cell damage and regeneration and the mediators involved in this process will be important in understanding the pathogenesis of human H5N1 disease.

## Competing interests

The authors declare that they have no competing interests.

## Authors' contributions

MCWC, RWYC and JSMP conceived the study, planned the overall experimental design and wrote the manuscript. MCWC and RWYC carried out the experiments and interpretation of results; CLY, CCH and KMY carried out experiments in the BSL-2 laboratory and assisted in experiments in the BSL-3 laboratory. WHC and CKL provided the lung biopsy specimens and JMN developed the methods of immunohistochemistry and lectin staining and YG critically reviewed the manuscript.
